# Mulberry leaves and their potential effects against cardiometabolic risks: a review of chemical compositions, biological properties and clinical efficacy

**DOI:** 10.1080/13880209.2018.1424210

**Published:** 2018-01-18

**Authors:** Thanchanit Thaipitakwong, Surawej Numhom, Pornanong Aramwit

**Affiliations:** a Bioactive Resources for Innovative Clinical Applications Research Unit and Department of Pharmacy Practice, Faculty of Pharmaceutical Sciences, Chulalongkorn University, Bangkok, Thailand;; b Department of Surgery, Faculty of Medicine, Plastic and Maxillofacial Surgery, Ramathibodi Hospital, Mahidol University, Bangkok, Thailand

**Keywords:** *Morus* spp., hyperglycaemia, hyperlipidaemia, obesity, hypertension, oxidative stress, cardiovascular diseases

## Abstract

**Context:** Cardiometabolic risks are regarded as the crucial factors associated with type 2 diabetes (T2DM) and cardiovascular diseases (CVD). Regarding an increased attention to medicinal plants in the current healthcare system, the effects of mulberry (*Morus* spp., Moraceae) leaves on cardiometabolic risks have been consecutively considered in scientific research.

**Objective:** The present review compiles and summarizes the chemical compositions, biological properties and clinical efficacy of mulberry leaves that are related to the amelioration of cardiometabolic risks.

**Methods:** Published English literature from the PubMed, Science Direct and Google Scholar databases was searched by using ‘mulberry leaves’ ‘*Morus* spp.’, ‘hyperglycemia’, ‘hyperlipidemia’, ‘obesity’, ‘hypertension’, ‘oxidative stress’, ‘atherosclerosis’ and ‘cardiovascular diseases’ as the keywords. The relevant articles published over the past two decades were identified and reviewed.

**Results:** Mulberry leaves contain numerous chemical constituents. 1-Deoxynojirimycin (DNJ), phenolics and flavonoids are the prominent functional compounds. Preclinical and clinical studies showed that mulberry leaves possessed various beneficial effects against cardiometabolic risks, including antihyperglycaemic, antihyperlipidaemic, antiobesity, antihypertensive, antioxidative, anti-inflammatory, anti-atherosclerotic and cardioprotective effects.

**Conclusions:** Mulberry leaves could be a promising therapeutic option for modulating cardiometabolic risks. However, further investigations should be performed to substantiate the potential of mulberry leaves in practical uses.

## Introduction

Cardiometabolic risks consist of glucose intolerance and/or insulin resistance, abdominal obesity, dyslipidaemia and hypertension. It is evident that cardiometabolic risks are the crucial factors potentiating the development of type 2 diabetes (T2DM), cardiovascular diseases (CVD) and the related morbidity and mortality (Fisher 2006; Brunzell et al. 2008). Oxidative stress, endothelial dysfunction and vascular inflammation are also associated with the underlying pathogenesis of CVD (Savoia et al. 2011). These abnormalities can be present in isolation or clustered together (Brunzell et al. 2008). Clinical evidence suggests that lifestyle changes, including nutritional therapy, exercise and smoking cessation, together with conventional medicines, are the cornerstones of successful management. A combination of therapeutic strategies can increase the effectiveness of treatment (Manrique et al. 2005; Fisher 2006).

Interestingly, a number of medicinal plants have been widely used as functional foods and alternative medicines for the prevention and treatment of several diseases. Plants are considered the potential resources of various bioactive compounds. Safety and efficacy of medicinal plants have been well-accepted as seen in long-term traditional uses and scientific research. Furthermore, plant therapies are more easily accessible and affordable compared to modern medicines. For these reasons, medicinal plants become an important part of the primary healthcare system nowadays.

Mulberry (*Morus* spp., Moraceae) is a well-known medicinal plant. White mulberry (*Morus alba*), black mulberry (*M. nigra*) and red mulberry (*M. rubra*) are the most notable species of the genus *Morus* (Yigit et al. 2010). Different species of mulberry are distributed in tropical, subtropical and temperate areas throughout the world. However, the majority of the plant is widespread in Asian countries, such as China, Japan, Korea and India (Sánchez 2000).

Mulberry is a multi-functional plant. Being an excellent source of nutrients and phytochemicals, mulberry has been established as functional food (Srivastava et al. 2006). The fresh fruits are edible and harvested for food production, such as juice, jam and jelly (Yigit et al. 2010). Meanwhile, the leaves are highly palatable (Srivastava et al. 2006). Mulberry leaves play a pivotal role in the sericulture industry because they serve as the sole food of silkworm (*Bombyx mori*) (Sánchez 2000). The leaves are also cultivated for dairy animal feed due to the positive effect on milk production (Gupta et al. 2005). Herbal tea made from mulberry leaves are consumed as a healthy beverage among Asian countries (Chan et al. 2016). In the folk remedies, various parts of mulberry tree, including root bark, leaves and fruits, have been traditionally used for the treatment of fever, cough, hyperlipidaemia, hypertension and hyperglycaemia (Chan et al. 2016). Mulberry leaves-derived products in the form of powders, extracts and capsules are now commercially available as functional foods and dietary supplements for controlling body weight and blood glucose.

Scientific studies suggest that mulberry leaves contain a cluster of bioactive compounds and possess several pharmacological effects. Nonetheless, evidence demonstrating beneficial effects of mulberry leaves against cardiometabolic risks remains scarce at present. Moreover, most of the prior studies focused on *M. alba*. Attention to the other species of mulberry has been limited in few studies. As a result, this review aims to compile and summarize the chemical compositions, biological properties and clinical efficacy of mulberry leaves that are related to the amelioration of cardiometabolic risks, regardless of the mulberry species and experimental models.

## Literature search

The English language literature available in the PubMed, Science Direct and Google Scholar databases was searched by using the keywords: ‘mulberry leaves’, ‘*Morus* spp.’, ‘hyperglycemia’, ‘hyperlipidemia’, ‘obesity’, ‘hypertension’, ‘oxidative stress’, ‘atherosclerosis’ and ‘cardiovascular diseases’. The relevant articles published over the past two decades were identified and reviewed.

## Chemical composition

Determination of chemical compositions showed that the ash and moisture content of mulberry leaves ranged between 8.19–12.63% and 72.16–79.35%, respectively (Adeduntan and Oyerinde 2010). Mulberry leaves are a precious source of macro- and micronutrients, and organic acids (Table 1). Overall, the leaves are rich in protein. The protein content found in mulberry leaves is significantly higher than other green leafy vegetables (Gupta et al. 2005). In addition, mulberry leaves consist of ascorbic acid and minerals. Calcium and potassium are the two most abundant elements, whereas sodium is present in less quantity (Yigit et al. 2010; Sanchez-Salcedo et al. 2017). Previous analysis also detected antinutritional components, including fibre, cyanide and tannin, in mulberry leaves in the range of 8.74–13.70%, 1.01–2.14 mg/kg and 3.54–5.32 mg/kg, respectively (Adeduntan and Oyerinde 2010).

**Table 1.** Chemical compositions of mulberry leaves.

Chemical compositions	Content	References
Crude protein	13.4–19.4%	(Sanchez-Salcedo et al. 2017)
	18.41–24.63%	(Iqbal et al. 2012)
	21.24–21.66%	(Adeduntan and Oyerinde 2010)
Total carbohydrate	47.27–56.42%	(Adeduntan and Oyerinde 2010)
Crude fat	4.24–6.57%	(Iqbal et al. 2012)
	5.31–8.02%	(Adeduntan and Oyerinde 2010)
Vitamin		
Ascorbic acid	0.97–1.49 mg/g	(Iqbal et al. 2012)
Minerals		
Nitrogen	2.1–3.1 g/100 g	(Sanchez-Salcedo et al. 2017)
Phosphorus	0.1–0.2 g/100 g	(Sanchez-Salcedo et al. 2017)
Potassium	1.2–3.9 g/100 g	(Sanchez-Salcedo et al. 2017)
Calcium	1.7–3.9 g/100 g	(Sanchez-Salcedo et al. 2017)
Sodium	0.01 g/100 g	(Sanchez-Salcedo et al. 2017)
Magnesium	0.5–1.4 g/100 g	(Sanchez-Salcedo et al. 2017)
Sulphur	0.2–0.3 g/100 g	(Sanchez-Salcedo et al. 2017)
Iron	119.3–241.8 mg/kg	(Sanchez-Salcedo et al. 2017)
Zinc	23.9–39.5 mg/kg	(Sanchez-Salcedo et al. 2017)
Manganese	35.8–90.5 mg/kg	(Sanchez-Salcedo et al. 2017)
Boron	253.5–825.3 mg/kg	(Sanchez-Salcedo et al. 2017)
Copper	4.2–5.9 mg/kg	(Sanchez-Salcedo et al. 2017)
Molybdenum	0.8–2.3 mg/kg	(Sanchez-Salcedo et al. 2017)
Nickel	1.7–5.4 mg/kg	(Sanchez-Salcedo et al. 2017)
Lead	0.3–0.8 mg/kg	(Sanchez-Salcedo et al. 2017)
Carbon	37.4–41.4 g/100 g	(Sanchez-Salcedo et al. 2017)
Lithium	1.9–17.2 mg/kg	(Sanchez-Salcedo et al. 2017)
Titanium	5.4–10.8 mg/kg	(Sanchez-Salcedo et al. 2017)
Organic acids		
Citric acid	32.2–105.5 mg/100 g	(Sanchez-Salcedo et al. 2017)
Malic acid	43.7–72.6 mg/100 g	(Sanchez-Salcedo et al. 2017)

Mulberry leaves contain iminosugar alkaloids, which exhibit an inhibitory effect on mammalian glucosidase enzymes. The most dominant compound is 1-deoxynojirimycin (DNJ) (Asano et al. 2000). Prior studies reported the concentrations of DNJ were 1.389–3.483 mg/g (Song et al. 2009) and 0.1341–1.472 mg/g (Hu et al. 2013) in dried leaves of mulberry from different varieties.

A number of antioxidative compounds were isolated in chemical analyses. Phenolic acids in mulberry leaves were identified as caffeic, gallic, protocatechuic, *p*-hydroxybenzoic, vanillic, chlorogenic, syringic, *p*-coumaric, ferulic and *m*-coumaric acids (Thabti et al. 2012). Meanwhile, the detected flavonol compounds were rutin (3-*O*-rutinoside quercetin), izoquercitrin (quercetin 3-β-d-glucoside) and astragalin (kaempferol 3-β-d-glucopyranoside) (Thabti et al. 2012; Flaczyk et al. 2013). Quantitative determination found that the values of total phenolics and total flavonoids in mulberry leaves were 16.21–24.37 mg gallic acid equivalent/g and 26.41–31.28 mg rutin equivalent/g, respectively (Flaczyk et al. 2013).

## Biological properties

### Antihyperglycaemic effect

A single-dose and long-term administration of mulberry leaves possessed beneficial effects on glycaemic outcomes in animal studies. Antihyperglycaemic action of mulberry leaves was mainly determined based on the outcomes related to postprandial glucose (PPG). A single administration of mulberry leaves significantly suppressed the peak level and the incremental area under the curve (iAUC) of glucose excursion after carbohydrate loading (Park et al. 2009; Kim GN et al. 2011). Meanwhile, long-term mulberry leaves ingestion tended to normalize the levels of fasting plasma glucose (FPG), glycated haemoglobin (HbA1c), fructosamine and insulin indexes of diabetic animals to nearly normal values (Mohammadi and Naik 2008; Park et al. 2009; Kim JY et al. 2011; Wilson and Islam 2015). This indicated that mulberry leaves could be effective to improve glycaemic control and reverse insulin resistance. Moreover, histological examination showed that mulberry leaves treatment restored the size and number of pancreatic β-cells of diabetic animals closely to the baseline levels (Mohammadi and Naik 2008).

DNJ is regarded as the most potent antihyperglycaemic compound of mulberry leaves (Hu et al. 2013). As DNJ and glucose have similar structures, DNJ competitively blocks the active site of polysaccharide-degrading enzymes in the digestive tract. When the enzymes are inhibited, digestion and absorption of dietary carbohydrates are eventually diminished (Asano et al. 2000; Asano 2003). *In vitro* studies demonstrated the inhibitory effect of a DNJ-concentrated fraction against enzymes in the α-glucosidase class and the strongest inhibition was seen on sucrase enzyme (Miyahara et al. 2004; Kim GN et al. 2011). The results additionally suggested that DNJ was effectively comparable to voglibose, a conventional drug with α-glucosidase inhibitory activity, with a 50% inhibitory concentration (IC_50_) values against sucrase of 0.015 and 0.029 µg/mL for DNJ and voglibose, respectively (Miyahara et al. 2004).

Besides the local effect in the digestive tract, mulberry leaves showed antihyperglycaemic properties at different sites of action. The extract of mulberry leaves modified the expressions of gene and protein involved in glucose homeostasis in hepatic cells. As shown in *in vitro* experiments, the activities of gluconeogenic enzymes (phosphoenolpyruvate carboxykinase (PEPCK) and glucose-6-phosphatase (G-6-Pase)) were suppressed (Liu et al. 2016), whereas the activities of glycolysis enzymes (glucokinase (GK), phosphofructokinase (PFK) and pyruvate kinase (PK)) were promoted in a dose-dependent manner (Li et al. 2013). Mulberry leaves extract also activated phosphatidylinositol-3-kinase (PI3K)/protein kinase B (AKT) and glycogen synthase kinase-3β (GSK-3β) signalling pathways (Kim JY et al. 2011) and elevated glucose transporter-4 (GLUT-4) translocation (Liu et al. 2015) in skeletal muscles and adipose tissues. These mechanisms could explain the amelioration of insulin resistance of target tissues. Nonetheless, due to the low intestinal absorption of DNJ, it is questionable about the type of bioactive compounds that exert the latter mechanisms of action of mulberry leaves. Phenolics were proposed as a candidate in previous evidence (Kim JY et al. 2011).

### Antihyperlipidaemic effect

Animals treated with mulberry leaves experienced the marked reductions in total cholesterol (TC), low-density lipoprotein-cholesterol (LDL-C) and triglycerides (TG) and an increase in high-density lipoprotein cholesterol (HDL-C) in blood circulation (Ann et al. 2015; Kobayashi et al. 2015; Wilson and Islam 2015; Chang et al. 2016; Tond et al. 2016). In addition to blood lipid profiles, hepatic lipid accumulation was attenuated by mulberry leaves. The number and size of lipid droplets in hepatocytes in the treatment group were significantly lower than the control group (Ann et al. 2015; Chang et al. 2016).

Both *in vitro* and *in vivo* experiments observed that DNJ, phenolics and flavonoids were associated with lipid-lowering effects of mulberry leaves through multiple mechanisms of action. The extract of mulberry leaves enriched with DNJ, quercetin and kaempferol activated the expressions of AMP-activated protein kinase (AMPK) and peroxisome proliferator-activated receptor (PPAR)-α, leading to the increase in β-oxidation of free fatty acid and lipid breakdown (Tsuduki et al. 2009; Kobayashi et al. 2015). Meanwhile, polyphenol-rich extract of mulberry leaves containing caffeic acid, quercetin and hydroxyflavin decreased lipogenesis by regulating the activities of fatty acid synthase (FAS), glycerol-3-phosphate acyltransferase (GPAT), sterol regulatory element-binding proteins (SREBP)-1c and liver X receptor (LXR) (Ann et al. 2015; Sun et al. 2015; Chang et al. 2016).

### Anti-obesity effect

Mulberry leaves suppressed body weight gain induced by chronic ingestion of high-fat diet. At the end of study, the final body weight of animals fed with mulberry leaves was lower than the control group (Ann et al. 2015; Chang et al. 2016). The weight of visceral adipose tissues and body fat mass also diminished (Tsuduki et al. 2009; Chang et al. 2016). The above results from anthropometric measurement were confirmed by microscopic analysis. It was demonstrated that mulberry leaves significantly lowered the number of adipocytes, as well as the number and size of lipid droplets in the cells (Yang et al. 2014; Chang et al. 2016). Moreover, a gradual elevation of circulating adiponectin level, which is the anti-adiposity cytokine, was observed after long-term ingestion of mulberry leaves (Tond et al. 2016).

Inhibition of adipogenesis was stated as the major mechanism of action. Regarding the western blot analysis, the lower expression levels of SREBP-1 and PPAR-γ, which are transcriptional factors of adipocyte differentiation, in the cells treated with mulberry leaves extract were detected (Yang et al. 2014; Ann et al. 2015; Chang et al. 2016). Expression of the key lipogenic enzymes, for example, FAS and acetyl-coenzyme A carboxylase (ACC), also markedly were declined by mulberry leaves (Chang et al. 2016). Furthermore, adipocyte apoptosis was enhanced by the extract in *in vitro* models, contributing to the decreases in mature and functional adipocytes (Ann et al. 2015). It was hypothesized that phenolics were responsible for anti-obesity effect of mulberry leaves (Ann et al. 2015; Chang et al. 2016).

### Antihypertensive effect

In animal studies, abnormally elevated systolic blood pressure (SBP), diastolic blood pressure (DBP), mean arterial pressure (MAP) and heart rate were normalized by mulberry leaves ingestion (Naowaboot, Pannangpetch, Kukongviriyapan, Kukongviriyapan, et al. 2009; Yang et al. 2012; Nade et al. 2013). An investigation of vascular reactivity revealed that mulberry leaves improved the responses of blood vessels to exogenous stimulators. The impaired reactivity of blood vessels, including diminished dilatation and increased constriction, were significantly restored to the normal levels after long-term treatment of mulberry leaves (Naowaboot, Pannangpetch, Kukongviriyapan, Kukongviriyapan, et al. 2009).

Mulberry leaves reduced blood pressure and heart rate by inhibiting angiotensin-converting enzyme (ACE). *In vitro* studies observed the lower activity of ACE after the cells were treated with mulberry leaves extract with the IC_50_ of 29.8 mg/mL (Yang et al. 2012). Additionally, mulberry leaves acted as a calcium channel blocker. A decrease in vascular contraction in response to phenylephrine indicated that mulberry leaves blocked the pathway of calcium entry into the cells (Nade et al. 2013). The other possible mechanism of antihypertensive action could be from γ-aminobutyric acid (GABA) content in mulberry leaves extract because the mean blood pressure of the treatment group reduced with a similar trend as the group receiving pure GABA (Yang et al. 2012).

### Antioxidative effect

Ability against free radical formation and oxidative stress-induced tissue damage of mulberry leaves was confirmed by several evaluation methods. Regarding the analyses of scavenging capacities against 1,1-diphenyl-2-picrylhydrazyl (DPPH) and 2,2′-azino-bis-(3-ethylbenzthiazoline-6-sulphonic acid) (ABTS^+•^), mulberry leaves extract possessed anti-oxidative properties ranged between 1.89–2.12 and 6.12–9.89 mM Trolox equivalent/g of dried leaves, respectively (Iqbal et al. 2012). The extract additionally showed the electron donation capacity by reducing Fe^3+^ to Fe^2+^ (Arabshahi-Delouee and Urooj 2007; Iqbal et al. 2012). The studies consistently found that mulberry leaves demonstrated anti-oxidative effect in a dose-dependent fashion. However, the effect was weaker than ascorbic acid and butylated hydroxytoluene (BHT) that were used as positive controls in previous experiments (Arabshahi-Delouee and Urooj 2007; Naowaboot, Pannangpetch, Kukongviriyapan, Kongyingyoes, et al. 2009).

Determination of thiobarbituric acid reactive substances (TBARS) revealed the inhibitory effect of mulberry leaves on lipid peroxidation. In *in vitro* models, the extract of mulberry leaves dose-dependently suppressed the formation of malondialdehyde (MDA), which is an end-product of lipid peroxidation (Arabshahi-Delouee and Urooj 2007). Also, long-term administration of mulberry leaves normalized the elevation of MDA in plasma and tissues of chronic diabetic animals. The significant difference between the treatment and the non-treated group was observed in these studies and efficacy of mulberry leaves seemed comparable to insulin therapy (Naowaboot, Pannangpetch, Kukongviriyapan, Kongyingyoes, et al. 2009; Naowaboot, Pannangpetch, Kukongviriyapan, Kukongviriyapan, et al. 2009).

Furthermore, the activities of enzymes involved in the antioxidative defence system, including glutathione reductase, glutathione peroxidase, glutathione-*S*-transferase and superoxide dismutase, were significantly promoted in diabetic animals fed with mulberry leaves extract (Andallu and Varadacharyulu 2003).

Phenolics and flavonoids were found to be excellent antioxidants. Previous data showed that the fractions of mulberry leaves extract containing the higher values of phenolic and flavonoid compounds exhibited the stronger antioxidative property (Iqbal et al. 2012; Flaczyk et al. 2013). This could be from the robust correlation between antioxidant concentration and efficacy. For example, the Pearson correlations (*r*) were 0.973 and 0.537, respectively, for the correlations of total phenolics with DPPH and ABTS^+•^ methods (Flaczyk et al. 2013). In comparison with other compounds, chlorogenic acid displayed the strongest action against oxidative stress (Iqbal et al. 2012; Sanchez-Salcedo et al. 2017).

### Anti-inflammatory effect

Mulberry leaves suppressed inflammatory processes via the signalling pathways of nuclear factor (NF)-κB, which is involved in macrophage activation-induced inflammation (Chao et al. 2013; Park et al. 2013). The effects were due to the decreases in proinflammatory cytokines, including inducible nitric oxide synthase (iNOS), cyclooxygenase-2 (COX-2), tumour necrosis factor-alpha (TNF-α), interleukin (IL)-1β and IL-6, contributing to the down-regulation of NF-κB transcription factors (Park et al. 2013). In addition, endothelial cell adhesion of monocytes induced by TNF-α was significantly reduced by mulberry leaves extract (Chao et al. 2013). Previous studies also revealed that mulberry leaves possessed anti-inflammatory effect with a dose–response relationship (Chao et al. 2013; Park et al. 2013).

### Anti-atherosclerosis

Mulberry leaves treatment attenuated the development of atherosclerotic events through several pathways. In *in vitro* experiments, mulberry leaves extract dose-dependently inhibited the oxidative modification of LDL particles and the LDL transfer through arterial wall in the process of foam cell formation. It was confirmed by analysis of intracellular lipid that lipid accumulation in foam cells significantly diminished (Yang et al. 2011). The growth curve assay also found that mulberry leaves extract was capable of inhibiting vascular smooth muscle cell (VSMC) proliferation and migration in cell lines (Chan et al. 2009, 2010) and animals fed with high-cholesterol diet in a dose-dependent manner (Chan et al. 2013). In addition, mulberry leaves treatment restored the levels of circulating markers of endothelial dysfunction, including soluble vascular cell adhesion molecule-1 (sVCAM-1), fibrinogen and nitric oxide, to the normal levels (Sharma et al. 2010).

Benefits of mulberry leaves were not seen only in the early stage of atherosclerosis, but were also effective even when atherosclerotic plaques were formed. Plaque volume was significantly decreased after the long-term treatment with mulberry leaves in previous animal studies (Chan et al. 2013).

### Cardioprotective effect

Cardiac structure and function were preserved by mulberry leaves administration in various experimental models. After daily treatment of mulberry leaves in animals, the results showed the amelioration of myocardial damage induced by isoproterenol (ISO). When compared to the non-treated group, the treatment group had the lower levels of elevated cardiac markers and the smaller area of myocarditis and myonecrosis (Nade et al. 2013). Similarly, mulberry leaves treatment was associated with the close-to-normal structure of myocardial tissues without much infiltration of inflammatory cytokines and fibrous tissues in the myosin-induced myocarditis models (Arumugam et al. 2012). In this study, the treatment also preserved cardiac hemodynamic function by reversing systolic and diastolic impairment of myocardium, indicating protective effect on the left ventricular remodelling development (Arumugam et al. 2012).

## Clinical efficacy

### Antihyperglycaemic effect

Therapeutic efficacy of mulberry leaves on blood glucose has been widely reported in clinical researches. In general, DNJ is established as the major active constituent of the mulberry leaves interventions and the amount of ingestion is defined based on the concentration of DNJ in the products. Evidence suggests the effective dose of DNJ for human ranges between 6 and 24 mg (Kimura et al. 2007). A single administration of DNJ-enriched mulberry leaves products attenuated postprandial hyperglycaemia during carbohydrate tolerance test. The peak level and the iAUC over time of PPG were significantly lowered in the mulberry-treated group compared to the control group (Kimura et al. 2007; Mudra et al. 2007; Asai et al. 2011; Nakamura et al. 2011; Chung et al. 2013; Banu et al. 2015). The main findings were consistent among participants with and without T2DM. Regardless of carbohydrate sources, a significant suppression of peak glucose level was initially noticed within 30 min after receiving the intervention (Asai et al. 2011; Chung et al. 2013). However, the higher dose of mulberry DNJ was required to effectively suppress elevated blood glucose when complex carbohydrates were consumed (Nakamura et al. 2009).

Daily administration of mulberry leaves products also had benefits on long-term glycaemic control. A 4-week ingestion of mulberry leaf capsules at a dose of 6 mg of DNJ/meal reversed postprandial responses to a high carbohydrate meal in prediabetic patients. Compared to the control group, those in the treatment group had improvement in insulin (*p* = 0.0207), and c-peptide (*p* = 0.0590) indexes after carbohydrate loading. However, the level of PPG seemed not to be affected by mulberry DNJ in this study (Kim et al. 2015). It might be because the dose of DNJ was too low to be effective. Meanwhile, daily supplementation with 6 mg/meal of mulberry DNJ for 12 weeks resulted in a significant improvement in 1,5-anhydroglucitol (1,5-AG) (*p* < 0.001), a robust marker of postprandial glycaemia, throughout the study period in participants with impaired glucose metabolism. Although no difference in FPG, HbA1c and glycated albumin between the groups was observed, the levels tended to be reduced when compared to baseline (Asai et al. 2011). Nonetheless, mulberry leaves supplementation had no effect in persons without diabetes. Mulberry leaves powder enriched with DNJ at the dose of 18 mg/meal did not cause a significant change in FPG among healthy volunteers throughout 38 days of the treatment period (Kimura et al. 2007). The possible explanations of this study results are that the study duration seemed too short and participants had no abnormal blood glucose level at baseline.

### Antihyperlipidaemic effect

Antihyperlipidaemic effect of mulberry leaves was determined in different populations. A 12-week single group study in patients with early-stage dyslipidaemia found that routine supplementation of mulberry leaves tablets containing 0.367 mg of DNJ/tablet significantly decreased TC (4.9%, *p* < 0.05), LDL-C (5.6%, *p* < 0.05) and TG (14.1%, *p* < 0.05) and increased HDL-C (19.7%, *p* < 0.05) levels when compared to the baseline levels (Aramwit et al. 2011). This study also proposed that the lipid-lowering efficacy of mulberry leaves was superior to lifestyle modification alone (Aramwit et al. 2011). The other 12-week single group study was designed to investigate the efficacy of mulberry leaves capsules corresponding to 36 mg of DNJ/day among patients with hypertriglyceridemia. The treatment had a moderate effect on TG, which was decreased from 312 ± 90 mg/dL at baseline to 252 ± 78 mg/dL at week 12 (*p* = 0.058), whereas no statistically significant improvement in the other lipids was found (Kojima et al. 2010). Mulberry leaves in the form of brewed tea were also effective. Daily consumption of 6 g mulberry leaves tea reduced TC (9.8%), TG (14.9%) and LDL-C (2.02%) in patients with elevated TC, LDL-C and/or TG after 8-week intervention period. However, statistical changes at the end of the study were found when compared to the baseline level but were not observed between the groups (Banchobphutsa 2012).

Interestingly, mulberry leaves could be considered in individuals who co-existed with diabetes and dyslipidaemia. Mulberry leaves products also improved serum lipid profiles in patients with T2DM who had abnormal lipid levels in a 4-week clinical trial. The significant decreases in TC (12%, *p* < 0.01), TG (16%, *p* < 0.01), LDL-C (23%, *p* < 0.01), very low-density lipoprotein cholesterol (VLDL-C) (17%, *p* < 0.01) and plasma free fatty acids (12%, *p* < 0.01) from baseline were reported (Andallu et al. 2001). In this study, the lipid-lowering effects of mulberry leaves were comparable to 5 mg/day of glibenclamide as the conventional therapy (Andallu et al. 2001). However, it should be noted that glibencamide, which is an antidiabetic drug, seemed not the most appropriate comparator for antihyperlipidaemic effect.

### Antioxidative and anti-inflammatory effects

Antioxidative and anti-inflammatory effects of mulberry leaves in human models have been investigated based on the measurement of surrogate markers. A previous clinical trial suggested a significant reduction in 8-isoprostane, a marker of oxidative injury, in patients with mild dyslipidaemia whose diets were supplemented with mulberry leaves tablet containing 0.367 mg of DNJ/tablet for the duration of 12 weeks. An improvement in glutathione peroxidase activities in erythrocytes was also observed. The mean monthly level of C-reactive protein (CRP) tended to decrease in this study. Nonetheless, no significant difference between the initial and the final levels of glutathione peroxidase activities and CRP was reported (Aramwit et al. 2013). The lack of significant change might result from the low baseline levels of the study population.

Additionally, determination of the end-products of lipid peroxidation observed that mulberry leaves reduced the level of peroxides in various biological samples, including plasma, erythrocyte and urine, after 4 weeks of the treatment (Andallu et al. 2001). Moreover, mulberry leaves were effective for reducing small dense-LDL (sd-LDL) particles (Kojima et al. 2010) and lipids on erythrocyte membrane (Andallu et al. 2001), which are strongly associated with lipid peroxidation, vascular membrane rigidity and atherosclerotic plaque formation.

## Discussion and conclusions

Mulberry leaves could be a promising therapeutic option for modulating cardiometabolic risks. Various nutrients and functional phytochemicals were found in mulberry leaves. The dominant compounds responsible for pharmacological effects on cardiometabolic risks of mulberry leaves included DNJ, phenolics and flavonoids. As shown in previous reports, we observed that the range of chemical compositions in the leaves can considerably vary among different samples. For mulberry leaves, species, parts of branches, harvesting seasons and planting regions were the factors influencing the amount of chemical compositions (Nuengchamnong et al. 2007; Song et al. 2009; Iqbal et al. 2012). As an inconsistent concentration of phytochemicals is the major constraint of natural-derived products, exploring the standardized methods to control phytochemicals quantity in mulberry leaves is the big challenge for future research.

The characteristics of included preclinical- and clinical studies demonstrating biological properties and clinical efficacy of mulberry leaves are summarized in the present review (Tables 2 and 3, respectively). According to preclinical experiments, mulberry leaves treatment attenuated cardiometabolic risks through multiple actions against hyperglycaemia, dyslipidaemia, obesity, hypertension, oxidative stress, atherosclerosis and structural- and functional heart defects. Nonetheless, it should be noted that there remain the other factors involved in cardiometabolic abnormalities, such as adipokines, lipoproteins and prothrombotic profiles. Effects of mulberry leaves on these mentioned factors are still unknown in the current research. Further investigations should be performed to better understand the additional effects of mulberry leaves.

**Table 2.** Preclinical studies demonstrating biological properties of mulberry leaves.

	Mulberry leaves interventions					
Models	Species	Solvent extracts	Preparation	Dose	Duration	References
*Antihyperglycaemic effect*						
Healthy rats	*M. alba*	Water	Dried powder dissolved in distilled water	1 g/kg	Single dose	(Kim GN et al. 2011)
(A) Healthy rats (B) Non-obese diabetic rats	*M. alba*	Water	Extract (0.16 g DNJ/100 g) included into the diet	3.75 g/kg (6 mg/kg DNJ)	Single dose	(Park et al. 2009)
(A) Healthy rats (B) STZ-induced diabetic rats	*M. alba*	Ethanol	ND	400 and 600 mg/kg/day (*ad libitum*)	5 weeks	(Mohammadi and Naik 2008)
(A) Healthy rats (B) Non-obese diabetic rats	*M. alba*	–	Dried powder of grinded leaves (0.05 g DNJ/100 g) included into the diet	25% of the diet (*ad libitum*)	8 weeks	(Park et al. 2009)
(A) Healthy rats (B) STZ-induced diabetic rats	*M. alba*	–	Brewed tea (0.25% and 0.50%)	*Ad libitum*	4 weeks	(Wilson and Islam 2015)
(A) High fat diet-induced overweight rats (B) High sucrose diet-induced overweight rats	*M. alba*	Water	Extract (0.365 ± 0.025 g/100 g) included into the diet	5% of the diet	6 weeks	(Kim JY et al. 2011)
*Antihyperlipidaemic effect*						
(A) Healthy rats (B) STZ-induced diabetic rats	*M. alba*	–	Brewed tea (0.25% and 0.50%)	*Ad libitum*	4 weeks	(Wilson and Islam 2015)
High cholesterol diet-fed rats	*M. alba*	Methanol	Extract dissolved in drinking water (0.1 and 1 mg/mL)	*Ad libitum*	4 weeks	(Kobayashi et al. 2015)
STZ-induced diabetic rats	*M. alba*	(A) Ethanol (B) Crude extract	(A) Solution (B) Dried powder included into the diet	(A) 600 mg/kg (B) 25% of the diet	6 weeks	(Tond et al. 2016)
High fat diet-fed mice	*M. alba*	(A) Water (B) Ethanol	Extract included into the diet	0.5 and 1.5% of the diet	6 weeks	(Chang et al. 2016)
High fat diet-induced diabetic mice	*M. alba*	Ethanol	Extract included into the diet	133 and 666 mg/kg	12 weeks	(Ann et al. 2015)
High fat diet-fed mice	ND	–	Dried powder of grinded leaves included into the diet	1 and 3% of the diet	5 days	(Sun et al. 2015)
*Anti-obesity effect*						
High fat diet-fed mice	*M. alba*	(A) Water (B) Ethanol	Extract included into the diet	0.5 and 1.5% of diet	6 weeks	(Chang et al. 2016)
High fat diet-induced diabetic mice	*M. alba*	Ethanol	Extract included into the diet	133 and 666 mg/kg	12 weeks	(Ann et al. 2015)
*Anti-hypertensive effect*						
STZ-induced diabetic rats	*M. alba*	Ethanol	Extract dissolved in distilled water	0.25, 0.50 and 1 g/kg/day	8 weeks	(Naowaboot, Pannangpetch, Kukongviriyapan, Kukongviriyapan, et al. 2009)
Spontaneously hypertensive rats	*M. alba*	Water	Dried powder	0.53, 1.05 and 5.26 g/kg	(A) Single dose (B) 8 weeks	(Yang et al. 2012)
*Antioxidative effect*						
STZ-induced diabetic mice	*M. indica*	–	Dried powder of grinded leaves included into the diet	25% of the diet	8 weeks	(Andallu and Varadacharyulu 2003)
STZ-induced diabetic rats	*M. alba*	Ethanol	Extract dissolved in distilled water	0.25, 0.50 and 1 g/kg/day	6 weeks	(Naowaboot, Pannangpetch, Kukongviriyapan, Kongyingyoes, et al. 2009; Naowaboot, Pannangpetch, Kukongviriyapan, Kukongviriyapan, et al. 2009)
ISO-treated rats	*M. alba*	Methanol	Extract suspended in distilled water	25, 50 and 100 mg/kg	3 weeks	(Nade et al. 2013)
*Anti-inflammatory effect*						
Murine macrophage RAW 264.7 cells	*M. alba*	Ethanol	–	1, 10 and 50 μg/mL	24 hours	(Park et al. 2013)
Human aortic endothelial cells	*M. alba*	Methanol–water	–	25, 50 and 100 μg/mL	1 hour	(Chao et al. 2013)
*Anti-atherosclerosis*						
J774A.1 macrophage cells	*M. alba*	(A) Water (B) Ethanol	–	0.005–10 mg/mL	–	(Yang et al. 2011)
A7r5 aortic VSMCs	*M. alba*	Water	Dried powder suspended in distilled water	0.05, 0.1, 0.2, 0.5 and 1.0 mg/mL	72 hours	(Chan et al. 2009)
A7r5 aortic VSMCs	*M. alba*	(A) Water (B) Methanol–ethyl acetate	Dried powder suspended in distilled water	(A) 0.5–2.0 mg/mL (B) 0.2–0.6 mg/mL	24 hours	(Chan et al. 2010)
STZ-induced diabetic rats fed with atherosclerotic diet	*M. rubra*	Water	Extract included into diet	100, 200 and 400 mg/kg	30 days	(Sharma et al. 2010)
High cholesterol diet-fed rabbits	*M. alba*	(A) Water (B) Methanol–ethyl acetate	Dried powder suspended in distilled water	1 and 2% extract	25 weeks	(Chan et al. 2013)
*Cardioprotective effect*						
ISO-induced myocardial infarction rats	*M. alba*	Methanol	Extract suspended in distilled water	25, 50 and 100 mg/kg	3 weeks	(Nade et al. 2013)
Myosin-induced myocarditis rats	*M. alba*	–	Powder included into the diet	5% w/w of the diet	3 weeks	(Arumugam et al. 2012)

ND: not defined; STZ: streptozotocin.

**Table 3.** Clinical studies demonstrating clinical efficacy of mulberry leaves.

		Mulberry leaf interventions						
Design	Populations (N)	Species	Solvent extracts	Preparation	Dose	Duration	Outcomes	References
*Antihyperglycaemic effect*								
Parallel, double-blinded, RCT	Healthy volunteers (24)	*M. alba*	Ethanol–water	DNJ-enriched powder (1.5% DNJ) dissolved in water	0.4, 0.8 and 1.2 g (equivalent to 6, 12 and 18 mg DNJ, respectively)	Single dose	PPG and postprandial insulin during sucrose tolerance test	(Kimura et al. 2007)
Parallel, double-blinded, RCT	Healthy volunteers (50)	*M. alba*	Water	Dried powder of extract (0.36% DNJ) dissolved in water	1.25, 2.5 and 5 g (equivalent to 4.5, 9 and 18 mg DNJ, respectively)	Single dose	PPG during maltose tolerance test	(Chung et al. 2013)
Cross-over, double-blinded, RCT	Patients with impaired glucose metabolism (baseline FPG 100–140 mg/dL) (12)	*M. alba*	Ethanol–water	DNJ-enriched powder (1.5% DNJ) in capsule	3, 6 and 9 mg DNJ	Single dose	PPG during high-carbohydrate meal tolerance test	(Asai et al. 2011)
Cross-over, double-blinded, RCT	(A) Healthy volunteers (10) (B) Patients with T2DM (10)	*M. alba*	ND	Extract dissolved in water	1 g	Single dose	PPG during sucrose tolerance test and breath hydrogen concentration	(Mudra et al. 2007)
Parallel, double-blinded, RCT	Patients with T2DM (48)	ND	–	Brewed tea	70 mL	Single dose	PPG during high-carbohydrate meal tolerance test	(Banu et al. 2015)
Parallel, single-blinded, RCT	(A) Healthy volunteers (10) (B) Patients with untreated T2DM (5) (C) Patients with T2DM and treated with sulfonylurea (5)	ND	ND	3.3 g of extract (0.77% DNJ) included into jelly	254 µg DNJ	Single dose	PPG, postprandial insulin, HOMA-IR and breath hydrogen concentration	(Nakamura et al. 2011)
Parallel, double-blinded, RCT	Healthy volunteers (12)	*M. alba*	Ethanol–water	DNJ-enriched powder (1.5% DNJ) dissolved in water	1.2 g (equivalent to 18 mg DNJ) thrice daily before meals	38 days	FPG and FPI	(Kimura et al. 2007)
Parallel, double-blinded, RCT	Prediabetic subjects (42)	*M. alba*	Water	Tablet (0.36% DNJ)	6 tablets (equivalent to 6 mg DNJ) thrice daily with meals	4 weeks	PPG, postprandial insulin and postprandial c-peptide	(Kim et al. 2015)
Cross-over, double-blinded, RCT	Patients with impaired glucose metabolism (baseline FPG 110–140 mg/dL) (76)	*M. alba*	Ethanol–water	DNJ-enriched powder (1.5% DNJ) in capsule	3 capsules (equivalent to 6 mg DNJ) thrice daily before meals	12 weeks	FPG, FPI, HbA1c, glycated albumin and 1,5-AG	(Asai et al. 2011)
*Antihyperlipidaemic effect*								
Repeated measures, single group study	Patients with mild dyslipidaemia (baseline LDL-C 140–189 mg/dL) (23)	*M. alba*	ND	Tablet (0.367 mg DNJ/tablet)	3 tablets thrice daily before meals	12 weeks	Plasma TC, LDL-C, HDL-C and TG	(Aramwit et al. 2011)
Repeated measures, single group study	Patients with hypertriglyceridemia (baseline TG ≥200 mg/dL) (10)	ND	ND	Capsule (4 mg DNJ/capsule)	3 capsules (equivalent to 12 mg DNJ) thrice daily before meals	12 weeks	Plasma TC, LDL-C, HDL-C, TG, adiponectin, leptin and apolipoprotein-B	(Kojima et al. 2010)
Parallel, open-labelled, RCT	Patients with dyslipidaemia (baseline TC ≥200, LDL-C 101–190 or TG ≥150 mg/dL) (46)	*M. alba*	–	Brewed tea	2 g	8 weeks	Plasma TC, LDL-C, HDL-C and TG	(Banchobphutsa 2012)
Parallel, open-labelled, RCT	Patients with mild T2DM (12)	*M. indica*	ND	Capsule (500 mg/capsule)	2 capsules thrice daily before meals	4 weeks	Plasma TC, LDL-C, HDL-C and TG Erythrocyte membrane TC, LDL-C, HDL-C, TG and phospholipid	(Andallu et al. 2001)
*Antioxidative and anti-inflammatory effects*								
Parallel, open-labelled, RCT	Patients with mild T2DM (12)	*M. indica*	ND	Capsule (500 mg/capsule)	2 capsules thrice daily before meals	4 weeks	Plasma-, erythrocyte membrane-, urine-peroxides	(Andallu et al. 2001)
Repeated measures, single group study	Patients with mild dyslipidaemia (baseline LDL-C 140–189 mg/dL) (25)	*M. alba*	ND	Tablet (0.367 mg DNJ/tablet)	3 tablets thrice daily before meals	12 weeks	Erythrocyte glutathione peroxidase activity, 8-isoprostane and CRP	(Aramwit et al. 2013)

ND: not defined; RCT: randomized controlled trial; HOMA-IR: homeostasis of model assessment of insulin resistance; FPI: fasting plasma insulin.

Similar to preclinical studies, clinical trials suggested therapeutic efficacy of mulberry leaves in different populations who have cardiometabolic abnormalities. The most outstanding benefits of mulberry leaves were for glycaemic and lipid outcomes. However, evidences reported varying degree of efficacy. A plausible explanation is the remarkable differences in mulberry leaves intervention in terms of species, solvent extracts, preparations, and dosage of administration among the studies. The exact dose of mulberry leaves is now still unclear. Moreover, mulberry leaves were used as a single intervention in almost of the studies. Effect of mulberry leaves as an add-on therapy to conventional treatment should be further demonstrated for the future practical uses.

In addition, limitations in the study protocol are hereby noted. Randomized, double-blind, controlled trial with the large sample size is rather scarce at present. Also, the study period seemed insufficient for evaluating the long-term efficacy and safety of mulberry leaves.
